# Detecting early-warning signals of type 1 diabetes and its leading biomolecular networks by dynamical network biomarkers

**DOI:** 10.1186/1755-8794-6-S2-S8

**Published:** 2013-05-07

**Authors:** Xiaoping Liu, Rui Liu, Xing-Ming Zhao, Luonan Chen

**Affiliations:** 1 Key Laboratory of Systems Biology, SIBS-Novo Nordisk Translational Research Centre for PreDiabetes, Shanghai Institutes for Biological Sciences, Chinese Academy of Sciences, Shanghai 200031, China; 2 Department of Mathematics, South China University of Technology, Guangzhou 510640, China; 3 Collaborative Research Center for Innovative Mathematical Modelling, Institute of Industrial Science, University of Tokyo, Tokyo 153-8505, Japan; 4 Department of Computer Science, School of Electronics and Information Engineering, Tongji University, China

## Abstract

**Background:**

Type 1 diabetes (T1D) is a complex disease and harmful to human health, and most of the existing biomarkers are mainly to measure the disease phenotype after the disease onset (or drastic deterioration). Until now, there is no effective biomarker which can predict the upcoming disease (or pre-disease state) before disease onset or disease deterioration. Further, the detail molecular mechanism for such deterioration of the disease, *e.g*., driver genes or causal network of the disease, is still unclear.

**Methods:**

In this study, we detected early-warning signals of T1D and its leading biomolecular networks based on serial gene expression profiles of NOD (non-obese diabetic) mice by identifying a new type of biomarker, *i.e*., dynamical network biomarker (DNB) which forms a specific module for marking the time period just before the drastic deterioration of T1D.

**Results:**

Two dynamical network biomarkers were obtained to signal the emergence of two critical deteriorations for the disease, and could be used to predict the upcoming sudden changes during the disease progression. We found that the two critical transitions led to peri-insulitis and hyperglycemia in NOD mices, which are consistent with other independent experimental results from literature.

**Conclusions:**

The identified dynamical network biomarkers can be used to detect the early-warning signals of T1D and predict upcoming disease onset before the drastic deterioration. In addition, we also demonstrated that the leading biomolecular networks are causally related to the initiation and progression of T1D, and provided the biological insight into the molecular mechanism of T1D. Experimental data from literature and functional analysis on DNBs validated the computational results.

## Background

**Figure 1:**
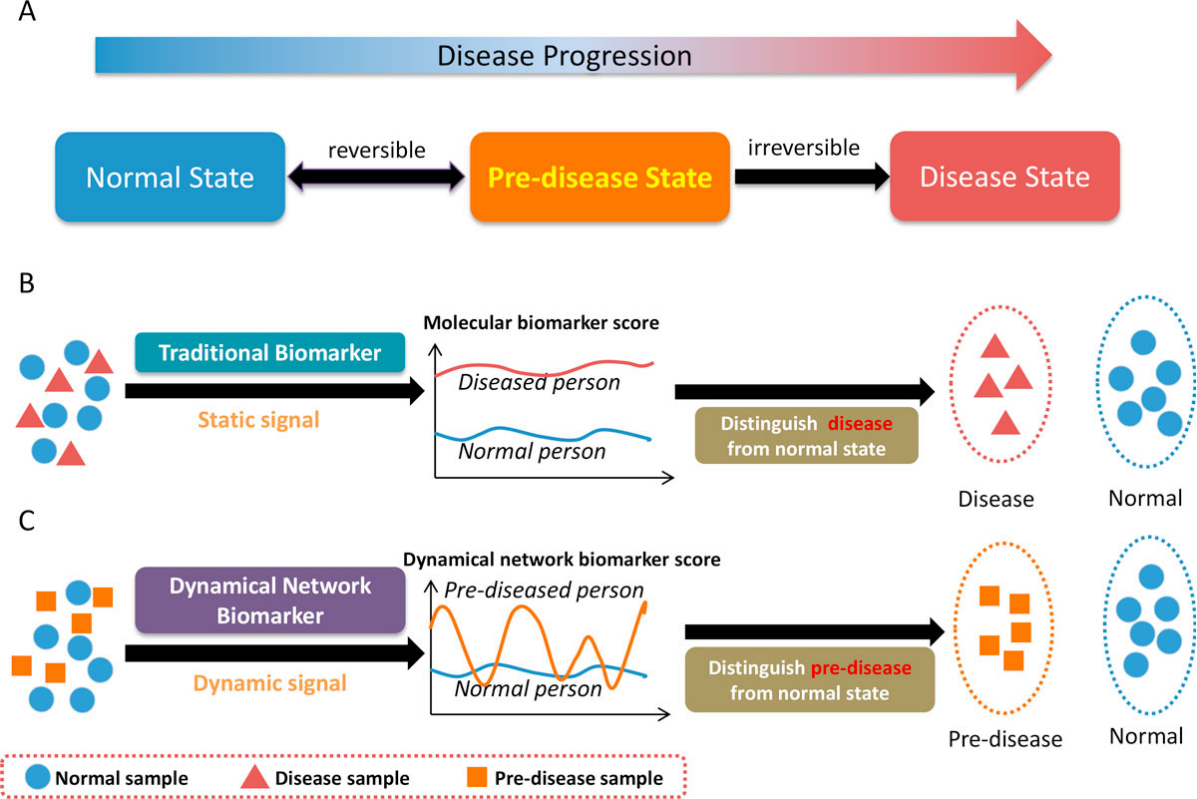
**Disease stages and disease biomarkers**. A). three stages during disease progression, *i.e*., normal state, pre-disease state and disease state. A normal state is a relatively healthy stage including the chronic inflammation period or the period during which the disease is under control, whereas a pre-disease state is the limit of the normal state just before the critical transition of the disease. At this stage, the pre-disease state is considered to be reversible to the normal state if appropriately treated. However, if the system passes over the critical point to the disease state, it usually becomes very difficult irreversibly to the normal state. B). Traditional biomarkers are indicators on the disease state and static measurements on the disease, and can distinguish disease samples from normal. C). The dynamical network biomarkers (DNBs) are signal of the pre-disease state and dynamical measurements on the pre-disease, and can distinguish pre-disease samples from normal samples, thus providing the early-warning signals for the pre-disease state.

Biomarkers or biological markers in biology are indicators of biological state for living organisms, which are objectively used to measure and evaluate normal biological processes, pathogenic processes, or pharmacologic responses to a therapeutic intervention. In medicine, a biomarker as an indicator is used to examine organ function or other aspect of health. However, traditional molecular biomarkers are usually used to examine only the current disease status of an organ based on the measurements of individual proteins or metabolites. It means that a traditional biomarker measures the disease state of an organ, after the organ has presented the characteristic of disease. In other words, it is to distinguish disease state from normal state, rather than early diagnosis. Generally, a disease progression can be divided into three stages, *i.e*., normal state, pre-disease state, and disease state [1], as shown in Figure 1A. A normal state is a relatively healthy stage including the chronic inflammation period or the period that the disease is under control, whereas a pre-disease state is the limit of the normal state just before the critical transition. At this stage, the pre-disease state is considered to be reversible to the normal state if appropriately treated. However, if the system passes over the critical point to the disease state, it becomes very difficult to be reversed to the normal state even by advanced medical treatment. Therefore, it is crucial to identify the pre-disease state (or achieve early diagnosis) so as to take the prevention action, which not only save the human lives but also medical resources. Hence, detecting pre-disease state or early-warning signal before the disease onset is more useful and important for the prevention of a disease. Although more and more computational and biological methods were developed to identify the biomarkers in different fields of disease [1-3], most of these biomarkers are mainly to distinguish disease state or phenotype from normal state, rather than to identify the pre-disease state, as shown in Figure 1B. Recently, a novel theoretical method has been developed to detect the pre-disease state before the disease state or onset [1], based not on traditional static biomarkers but on a new type of dynamical network biomarkers (DNBs). Because of the early-warning signal of the pre-disease state, a DNB (a group of molecules, *e.g*., genes, RNAs, proteins or metabolites) can directly be used to early diagnosis of the disease. In addition, DNB is of significance from both biological and medical viewpoints since it has been proven to be the leading network of the disease, which makes the first move from the normal state to the disease state, and therefore is strongly related to the driver genes or causal genes of the disease. As shown in Figure 1C, DNB is a new concept and indicator which has strong fluctuations among pre-disease samples, completely different from the conventional biomarkers which are required to keep consistent values for the respective disease and normal samples.

Type 1 diabetes (T1D) is a form of diabetes mellitus that is a clinically heterogeneous group of glucose intolerance syndromes, and usually has an autoimmune T cell-mediated etiology in which the pre-diabetic state is characterized by development of autoantibodies against certain proteins expressed by β cells, including insulin [4,5]. T1D is a complex disease and threatens the human health in the world, and the prediction of early-warning signals for T1D before the disease onset has not been reported. Also, the detail molecular mechanism for the disease progression, *e.g*., driver genes or causal network of the disease, is still unclear.

The non-obese diabetic (NOD) mouse strain [4-7] is a useful and important model of autoimmune disease and also an excellent tool for understanding the onset mechanism of T1D. The pancreatic lymph nodes are an important organ to preserve the antigen-specific T cell and surround the pancreatic islet [8,9]. And many reports have considered that they are related to T1D [4,6] and a number of research works used them to identify the potential biomarkers of T1D. In this study, instead of traditional molecular biomarker, we identify the pre-disease state of T1D by the new type of biomarker, *i.e*., dynamical network biomarker which forms a specific module of molecules (*e.g*., genes, RNAs, proteins, or metabolites) for marking the time period just before the drastic deterioration of T1D. Specifically, we detected early-warning signals of T1D and its leading networks based on the serial gene expression profiles of pancreatic lymph nodes in NOD mice by identifying two dynamical network biomarkers (DNB) in two different time points. By the theory of early-warning signals of complex diseases [1], a dynamical network biomarker will form a specific module in the pre-disease stage or near critical point before disease phenotype occurrence. Hence, the two critical points which were identified by the two respective DNBs would correspond to the early stages of disease onset for T1D. Actually, it is consistent with the reports about the pre-disease stage or disease onset time point of NOD mouse in the public references [5,6]. In addition, the two DNBs are also the leading networks, which are closely related to the driver molecules of the disease progression. Therefore, the two DNBs not only can be used to predict the upcoming disease onset before the drastic deterioration of the T1D phenotype, but also can reveal the molecular mechanism on the disease initiation and progression. Moreover, the computational results and the leading networks were also validated by experimental data from literature and functional analysis of the two DNBs. In particular, we found that the two DNBs could affect several famous pathways in T1D, such as "T cell receptor signaling pathway", "NF-kappa B signaling pathway" and "Insulin signaling pathway", on the pre-disease stages of the disease onset, which are all consistent with the existing results and other independent experimental results of literature.

## Methods

### Gene expression profiles

The non-obese diabetic (NOD) mouse is a useful and important model for autoimmune T1D. The pancreatic lymph nodes are the site of islet-cell-specific self-antigen presentation and important for the development of T1D. The gene expression profiles of pancreatic lymph nodes for T1D were obtained from GEO database (ID: GSE15150). The dataset includes the expression profiles of pancreatic lymph nodes of 35 female NOD mice samples at 6 different time points (10 days (7 samples), and 4 weeks (6 samples), 8 weeks (4 samples), 12 weeks (7 samples), 16 weeks (6 samples), and 20 weeks of age (5 samples)).

The original data was normalized by the logarithm ratios: Log10 (NOD processed signal / control signal), but this ratio cannot be directly used to calculate the correlation between genes. So the normalized data was transformed back to the general ratio (NOD processed signal / control signal) by exponent 10 operation.

### Criteria for DNB or the leading network

DNB can be detected from high throughput data (*e.g*., gene expression, protein expression, or metabolite expression data) based on three conditions, which are both theoretically and numerically proven [1]. Specifically, when the system approaches the critical point or pre-disease state, a dominant group or DNB appears and these DNB (or dominant group) members satisfy the following three conditions.

1. Pearson correlation coefficients (PCCs) between any pair of members in DNB become very high (*i.e*., drastic increase).

2. PCCs between one member of DNB and any other molecule of non-DNB become very low (*i.e*., drastic decrease).

3. Standard deviation (SD) for any member of DNB becomes very high (*i.e*., drastic increase).

Actually, it can also be shown that SD for any member of non DNB, and the PCCs between non-DNB members have no significant change. Clearly, the molecules in the dominant group are strongly and dynamically correlated in the pre-disease state. These molecules in the dominant group are expected to form a subnetwork or functional module from a network viewpoint, *i.e*., DNB. Therefore, the three conditions are considered as three criteria to detect the DNB or early-warning signals of the pre-disease state. Besides, since the three criteria are in fact the generic properties of the DNB members in dynamics whenever the system approaches a critical tipping point, these properties should lie in many complex diseases with sudden deterioration phenomena. The three measurable conditions can be summarized into a single index to detect the DNB, *i.e*., eqn.(1) as described next in details. Note that DNB is also the leading network, which makes the first move into the disease state from the normal state, and therefore, is causally related to the initiation and progression of the disease [1].

### Hierarchical clustering

**Figure 2:**
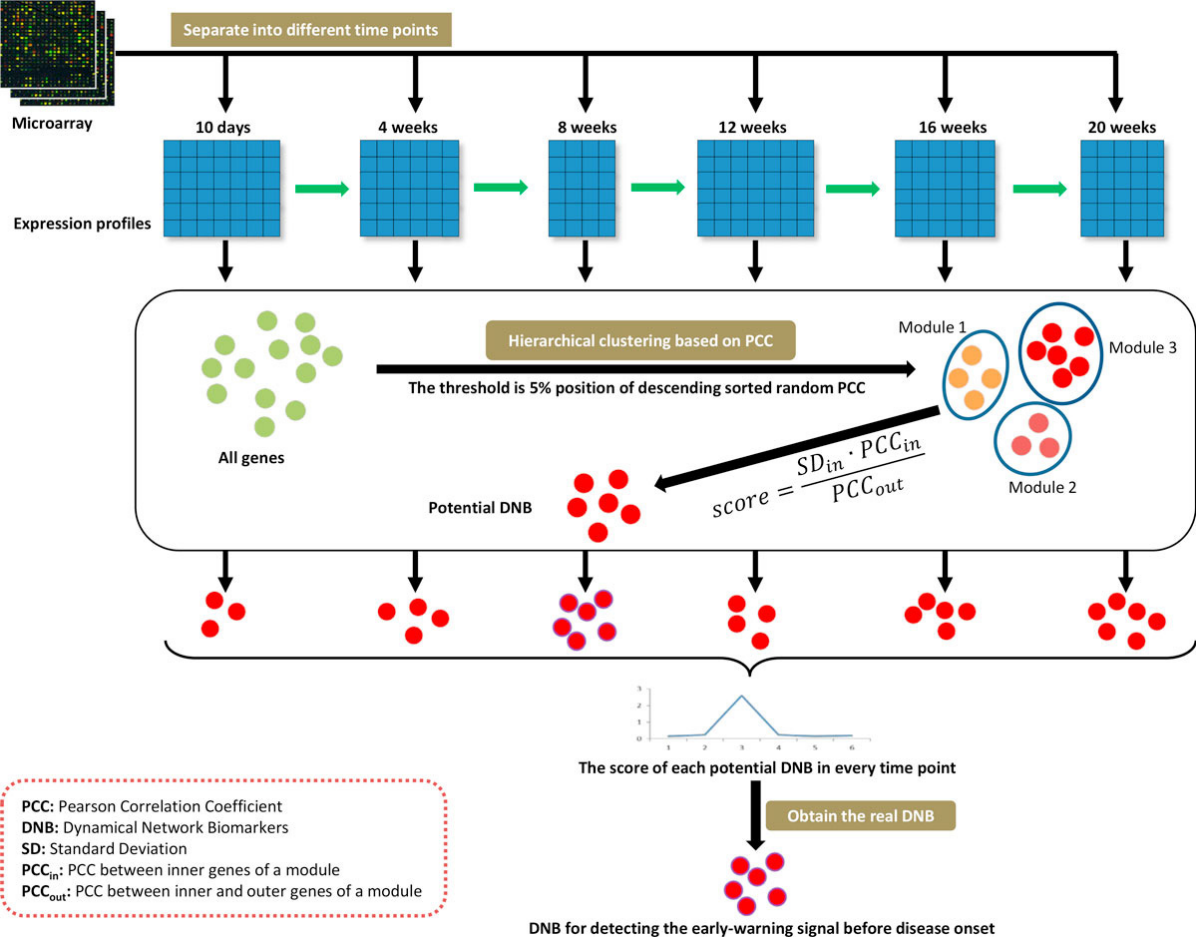
**Framework for identifying the dynamical network biomarkers by gene expression data**.

In every time point, the gene expression data was used to produce the modules or candidate DNBs by hierarchical clustering based on the distance of Pearson Correlation Coefficient (PCC), according to the three conditions of the DNB. In the hierarchical clustering, two modules can be combined into a new module, only if the average PCC inside the new module was greater than threshold PCC. The threshold also was used to control the end of clustering (Figure 2). For balancing the different sample size in every time point, we utilized an empirical randomization method to choose thresholds of high PCC in hierarchical clustering. Firstly, two random number vectors with the same length of the sample size in this time point were randomly produced and then the PCC between them was calculated; secondly, the random process was repeated 100,000 times and all of the PCCs were sorted by descending order; Thirdly, the PCC at the 5% position of the 100,000 descending order PCCs was regarded as the threshold for high PCC in this time point, and the average of 100,000 PCCs can be considered as the basic PCC value in this time point.

### Identification of dynamic network biomarkers (DNB)

The dynamic network biomarkers (DNB) were identified by a new method [1,17] which can be utilized to detect the early-warning signals before the onset of T1D, according to the three conditions. The three conditions are summarized into a single index or score (*i.e*., a composite index) by the following formula:

(1) $$s=\frac{SD_{in}\cdot PCC_{in}}{PCC_{out}},$$

where  s is the score of a module or a candidate DNB, $SD_{in}$is the average standard deviation of genes expression in the module, $PCC_{in}$ is the average Pearson correlation coefficient among genes inside of the module, and $PCC_{out}$ is the average Pearson correlation coefficient between inside and outside genes of the module. Clearly, (1) represents the three conditions of the DNB.

For every time point, the score of every module was calculated by the above formula based on the gene expression of the module in this time point and the best module with the highest score was regarded as the potential DNB in this time point. Then, these identified potential DNBs in every time point were compared each other, and the highest score DNB in all time points was the DNB for detecting the early-warning signals before the disease onset (Figure 2). The time point corresponding to the DNB was called critical point, which is the early stage of the disease onset. Also, the DNB is the leading network, which leads the system to the disease state.

### Regulated gene of the DNB

The regulated genes by the identified DNB module are picked up from the onset time point. The genes, which are highly correlated with DNB module in onset time point and are also differential expression genes between the critical point and onset time point, are regarded as regulated genes by the DNB module. If a gene is highly related with at least 10 genes of DNB, we deem that the gene is highly related to the DNB module. Here the threshold of high relation is set to 0.05 of P-value of PCC and the threshold of differential expression is 0.05 of P-value of student's t test.

### Functional analysis of the DNB

The confidence of the identified DNB which is associated with early-warning signals before the disease onset can be proven by the evidence of disease phenotype from published references. The genes in the identified DNB have been linked and correlated to some pathways of KEGG (http://www.genome.jp/kegg/), and these pathways can be related to the disease initiation and progression. First, the genes of the DNB were mapped to pathways by the KEGG Mapper tools (http://www.genome.jp/kegg/mapper.html) which are the online tools for KEGG mapping. Subsequently, the correlations between the DNB and each pathway in KEGG were calculated in two time points that are the critical point and the disease onset point.

## Results

### Potential DNBs in every time point

**Figure 3:**
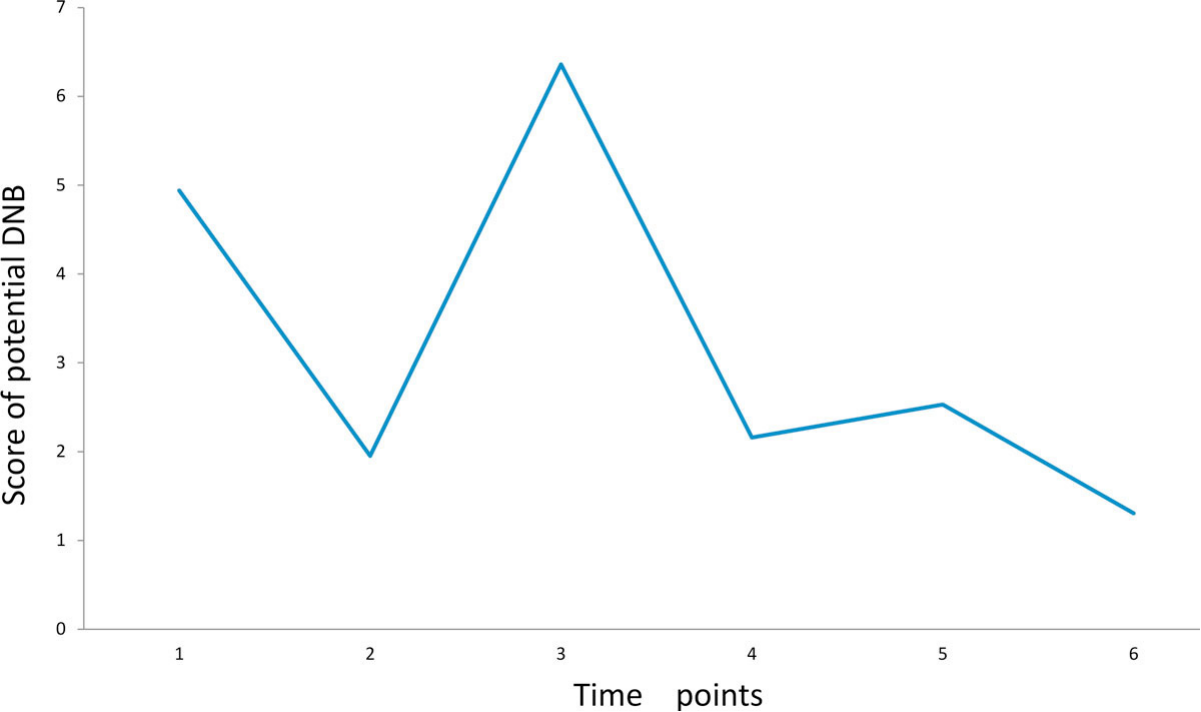
**Scores of potential DNBs in every time point**. The scores in the first and third time points are obviously higher than other time points, and therefore, the molecule modules in the first and the third time points are considered as the DNBs, which signal the drastic deteriorations of the disease during the progression of T1D.

Based on the criteria of DNB for PCC, we conducted the hierarchical clustering. In every time point, the genes were divided into different groups by hierarchical clustering based on expression data in this time point. The PCC was used as the distance of the hierarchical clustering, and a threshold can be used to control the end of the clustering. For balancing the different sample size for gene expression profiles in every time point, we utilized an empirical randomization method to choose thresholds of high PCC at the same level in hierarchical clustering. We chose the 5% position of sorted random PCC values, and it means that there is only 5% probability to randomly obtain such a high PCC value. In every time point, every module's score was calculated based on the expression data in this time point by formula (1), and the module of the highest score was regarded as a candidate or potential DNB in this time point. Therefore, we got 6 potential DNBs in 6 different time points and the scores of the potential DNBs were shown in Figure 3. We found that the scores of potential DNBs in the first (10 days) and the third (8 weeks) time point were obviously higher than other 4 time points, so the two potential DNBs in the first and the third time points were considered the real DNBs for signaling the drastic deteriorations of the disease during the progression of T1D. The first DNB in the first time point included 95 genes (Additional file 1) that were enriched to the biological processes of "regulation of cellular process", "cellular nitrogen compound metabolic process", "ncRNA processing" and so on by DAVID online tools (http://david.abcc.ncifcrf.gov/). The second DNB in the third time point included 96 genes (Additional file 1) that were enriched to the biological processes of "apoptosis", "programmed cell death", "cell death" and so on. The apoptosis of pancreatic β-cell plays an important role in the development of insulin deficiency and the onset progression of T1D, and the cell death of pancreatic β-cell also causes insulin deficiency and leads to hyperglycemia to trigger to diabetes [10,11]. The second DNB is early-warning signals of 12 weeks which is the time point of onset of T1D with hyperglycemia in female NOD mice [6,12]. Hence, the functional enrichment of the second DNB is consistent with not only the reports of existing literature, but also the phenotype of glycemic change.

### Early-warning signals of DNB in T1D development

**Figure 4:**
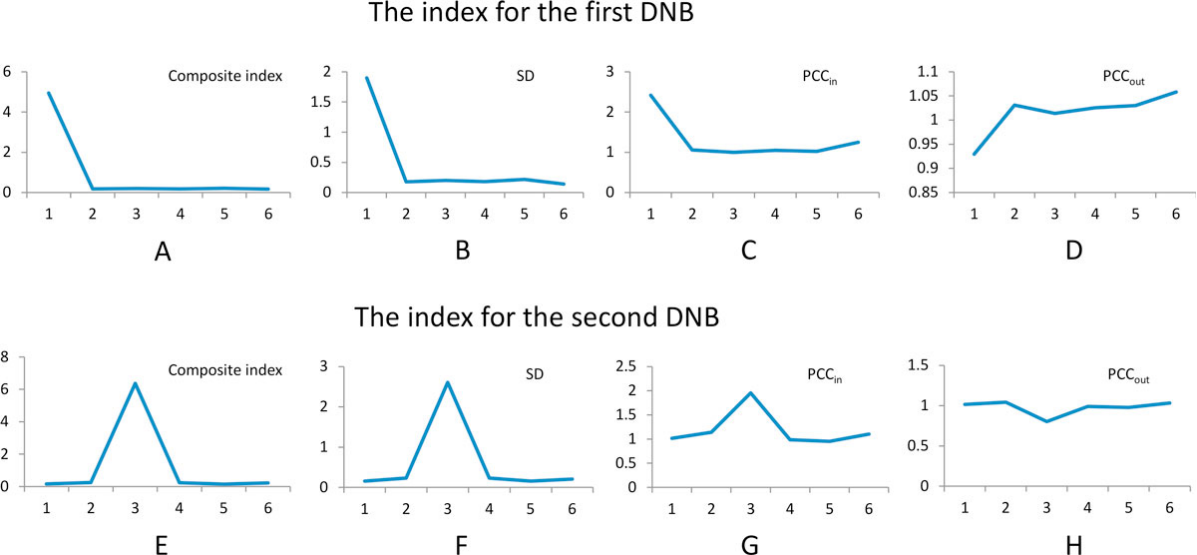
**The criteria of early-warning signals for T1D in NOD mice**. A) is the composite index for the first DNB in first time point, which combines the three conditions of DNB, *i.e*., B) standard deviation (SD), C) inner Pearson correlation coefficient (PCC_in_) and D) outer Pearson correlation coefficient (PCC_out_). E) is the composite index for the second DNB in third time point (which also combines the three conditions of DNB, *i.e*., F) SD, G) PCC_in _and H) PCC_out_). For balancing the different sample size for gene expression profiles in every time point, each Pearson correlation coefficient in the figure was divided by the average value of 100,000 times repeated randomly Pearson correlation coefficient with same sample size in this time point.

The two identified DNBs were exhibited by the score of module in every time point of T1D development, shown as Figure 4. From Figure 4 A-D, we can see that the first (10 days) time point was the critical time point of the first DNB, so the disease onset will appear at the next time point (4 weeks) of the critical point. In the critical point, the genes in the DNB module were strongly fluctuated with high deviation in their gene expression, were highly correlated with each other inside the module, and were lowly correlated with the genes not in the DNB module. Such a clear early-warning signal will appear at the critical point, at which that the genes in the DNB module are closely interconnected each other and hardly connected with those genes not in the DNB module. The disease phenotype of T1D would present after the critical time point. It is consistent with the report of public references about the development of T1D in female NOD mice [5,6,12]. The T cell initiation and lymphocytic infiltration would occur in the 4 weeks of age for female NOD mice [12], and the immune cell infiltrates surround the islet (peri-insulitis) at approximately 3 to 4 weeks of age in NOD mice [5,6]. Clearly, the first (10 days) time point is the critical point and the DNB can be used to detect the early-warning signals of the phenotype of peri-insulitis on the initiation stage of T1D.

From the score of the second DNB in every time point (Figure 4 E-H), we can see that the third (8 weeks) time point had the high score which can be the critical time point of the DNB module. Because the disease onset will appear at the next time point of the critical point, the phenotype of disease onset would present at the fourth time point (12 weeks). It is consistent with the reports of public reference and some biological experiments about the development of T1D in female NOD mice [5,6,12,13]. Disease onset of diabetes usually occurs at 12 to 14 weeks of age in female NOD mice[6], and destructive insulitis, leading to overt hyperglycemia, occurs around 12 weeks of age or later [5,12,13]. It validates that the third (8 weeks) time point is the critical point and this DNB can be utilized to detect the early-warning signal of phenotype before the disease onset of T1D.

### Molecular mechanism of DNB

For analyzing the mechanism of DNB during the development of T1D, the genes of the first DNB were mapped to the pathways of mouse in KEGG, and only 12 genes were identified from 21 pathways in KEGG. It means that many genes in the DNB would take part in more than one pathway and could affect the cross-talking among different pathways. In these pathways, the "Insulin signaling pathway" is an important one that regulates many metabolism and signal pathways, and is also associated with T1D development. It also includes some virus related pathway in the DNB mapped pathways, such as "Epstein-Barr virus infection". It is consistent with the theory which considers that T1D is a virus-triggered autoimmune response [14].

The genes of the second DNB were also mapped to the pathways of mouse in KEGG, and 11 genes were identified from 23 pathways in KEGG. There were also many genes which took part in more than one pathway, and they could affect the cross-talking among different pathways. There were three immune related pathways in which the genes of the second DNB participate, such as "T cell receptor signaling pathway", "NF-kappa B signaling pathway" and "Intestinal immune network for IgA production". Because T1D is an autoimmune disease and pancreatic lymph nodes are a major tissue to preserve T cell in pancreas, the "T cell receptor signaling pathway" would play an important role in the disease onset of T1D. The "NF-kappa B signaling pathway" is an important pathway in the T cell autoimmune and related to the onset of T1D [13,15].

T1D is a form of diabetes mellitus that results from autoimmune destruction of insulin-producing beta cells of the pancreas, and usually has an autoimmune T cell-mediated process in which insulin would be against by autoantibodies [4]. "T cell receptor signaling pathway", "NF-kappa B signaling pathway" and "Insulin signaling pathway" are three important pathways which are related to the disease onset of T1D. The correlation between the DNB and pathways was calculated by counting the number of high correlation pairs between genes in DNB and in pathway based on the threshold of 5% position of descending order random PCC. From the critical point to disease onset point, the number of high correlation gene pairs between DNB and different pathways was shown in Table 1. We can see that there are more genes of the pathway to correlate with DNB genes in disease onset point than critical point. It is possible that there are few relationships between DNB and pathways in the critical point, but many genes of DNB would take part in the regulation process for these important pathways in the disease onset point.

**Table 1:** Number of genes and interaction pairs with high correlations between DNB and pathways.

Pathway name	Time points	The first DNB			The second DNB		
		
		DNB	pathway	pairs	DNB	pathway	pairs
T cell receptor signaling pathway	Critical point	93	32	493	96	7	327
	Disease onset point	89	105	677	93	107	844

NF-kappa B signaling pathway	Critical point	94	28	372	96	9	425
	Disease onset point	92	87	588	89	87	705

Insulin signaling pathway	Critical point	95	31	606	59	5	152
	Disease onset point	94	133	839	93	134	1023

The column item "DNB" represents the number of genes in DNB which are highly correlated with the pathway in corresponding time point, the item "pathway" denotes the number of genes in the pathway which are highly correlated with the DNB in corresponding time point, and the item "pairs" means number of gene pairs which are highly correlated between genes separately in DNB and pathway in the corresponding time point.

### The regulated genes by DNB

**Figure 5:**
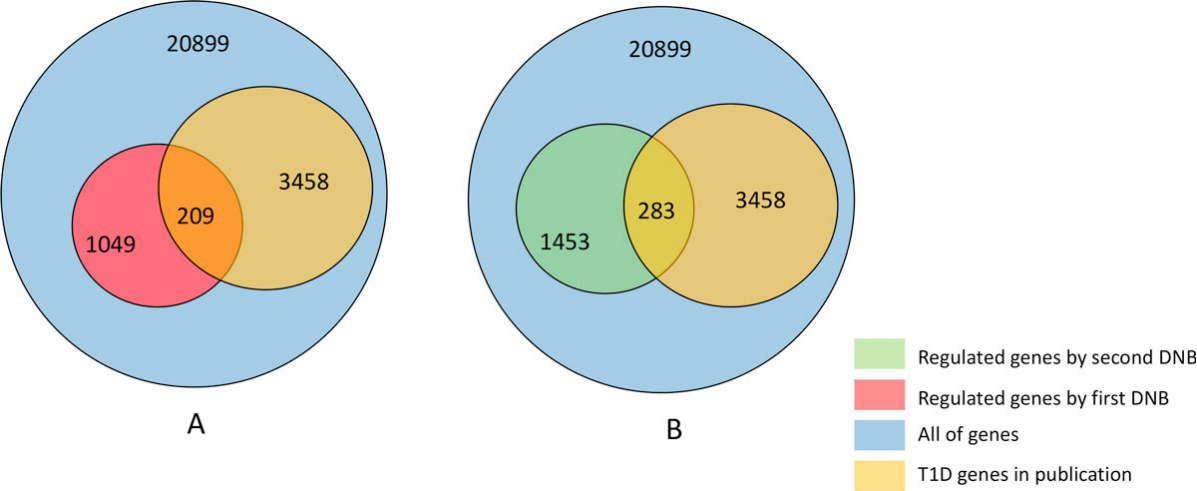
**The distribution of the regulated genes by DNB and T1D related genes in existing databases and publication literatures**. A) The distribution of regulated genes by the first DNB and T1D related genes, and B) the distribution of regulated gene by the second DNB and T1D related genes.

If the DNB can modulate the T1D onset in next time point after critical transition, the regulated genes by DNB should be T1D related genes. Hence, the regulated genes by two DNBs were separately picked up, and the T1D related genes were identified based on publication literature by T1Dbase website (http://www.t1dbase.org/). Finally, 20899 genes were mapped to T1Dbase database from 21000 genes of NOD mouse genome, and 3458 of these genes were identified as T1D related genes based on existing databases and publication literature. The number of the regulated genes by two DNBs were separately 1049 (additional file 2) and 1453 (additional file 3), and 209 and 283 of these regulated genes were identified as T1D related genes (Figure 5). Hypergeometric distribution was used to test the enrichment of regulated genes in the T1D related genes, and P-value of the regulated genes by the first DNB is 0.00177 and by the second DNB is 0.0012. We can see the regulated genes by two DNBs are significantly enriched to T1D related genes, and therefore, the two DNBs are really early-warning signals for development of T1D.

### Bridge between two DNBs

In the regulated genes by the first DNB, we found that 4 genes ('2410003K15RIK', 'TIMM50', 'HOXA4', 'LIN28A') are also in the second DNB, so it is possible that the second DNB can be affected by the first DNB and there is a bridge link between the two DNBs. We picked up the high correlation genes with the second DNB from the regulated genes by the first DNB, and differential expression was used to filter the selected genes. Finally, 110 genes (additional file 4) ware identified to link the two DNBs. Function enrichment analyses of the 110 genes showed that function of these genes was enriched to "mitochondrion" and "electron transport". Mitochondria are important subcellular fractions for cell apoptosis and play a crucial role in regulating cell death, and apoptosis can lead to hyperglycemia and trigger to diabetes onset [10,11]. The electron transport in mitochondrion is an important process for redox reactions and oxidative stress which can cause to islet cell autoimmunity and lead to T1D [16]. Hence, it is possible that the deterioration process from peri-insulitis to diabetes (hyperglycemia) is linked by the redox reactions and oxidative stress based on mitochondrion electron transport, and dysfunction of mitochondrion electron transport makes the disease worsening.

## Discussion

During the development of T1D, the disease progression will pass a pre-disease stage which is a critical transition period from normal stage to disease stage. After it passes through the critical point, the disease progression is generally irreversible [1]. As a novel biomarker, dynamical network biomarker (DNB) was developed to detect such a critical point just before the disease onset. In this study by proposing a new computational algorithm, we identified two DNB modules which can be separately used to predict the peri-insulitis on the early stage of diabetes and diabetes onset with overt hyperglycemia.

We consider that there are two kind of potential mechanisms for the DNB triggering the disease deterioration or phenotype change. On the one hand, the genes in the DNB were gathered together in the critical point, so they could interact and affect one another. Because most genes in DNB take part in more than one pathway, so these interactions and effects could make these genes deviate from the major pathway and regulate the disease related pathway together in disease onset point. For example, the genes in the two DNB modules were highly correlated with the three pathways on Table 1 in the disease onset point. It is possible that the genes in DNB regulate and control these pathways and make them move to disease phenotype. The "MAPK signaling pathway" is one of important pathways to regulate the glycogen and glucose metabolism and contains 267 known genes in KEGG database. In the critical point of the second DNB, there were only 15 genes of "MAPK signaling pathway" correlated with the DNB, and in the following time point, there were 260 genes of "MAPK signaling pathway" (additional file 5) correlated with the DNB. It means that there were more genes of "MAPK signaling pathway" to be regulated by the same DNB module in the disease onset point. It is possible that there were also stronger regulation effects of the DNB module to the metabolism of glycogen and glucose in the disease onset point. Although most of the genes in DNB belong to different pathways or metabolic processes, finally, these genes would induce these different pathways to the same disease phenotype in the disease onset point.

On the other hand, the genes in the DNB were gathered together to form a module by upstream signal in the critical point, the module can also regulate a small number of genes in some pathway in the critical point. These genes which were mediated by the module in the critical point could play important role in corresponding pathways and trigger the change of the disease phenotype in the disease onset point. For example, we can see from Table 1 that all genes in the second DNB modules were highly correlated with only 7 genes in the "T cell receptor signaling pathway" in the critical point. However, the 7 genes in the "T cell receptor signaling pathway" are located in the up- and middle-stream of this pathway in KEGG database, so it is possible that the 7 genes are the important or driving factors for the pathway to trigger disease phenotype change in the disease onset point. Actually, the DNB has been proven to be the gene group, which makes the first move from the normal state to the disease state. The peri-insulitis is the early-stage of T1D in NOD mice, and the link from peri-insulitis to diabetes is interesting for understanding the disease deterioration process. From the bridge of two DNBs, we can see the possible process that mitochondrion electron transport induces the apoptosis function of the second DNB and pushes the peri-insulitis to diabetes (hyperglycemia).

## Conclusion

Traditional biomarkers are usually used to distinguish disease state from normal state, rather than pre-disease state [18-20]. It means that a traditional biomarker measures the disease status of an organ, after the organ has presented the characteristic of disease [21-24]. In this study, completely different from the traditional molecular biomarkers, we distinguished the pre-disease state from the normal state by a new type of biomarkers, *i.e*., dynamical network biomarkers. Specifically, we found two dynamical network biomarkers which can be used to detect the early-warning signals and predict the upcoming disease onset of T1D by the theory of early-warning signals of complex disease [1,17]. Based on the three conditions of DNB, the two dynamical network biomarkers identified two respective critical time points which are the pre-disease stages of diabetes onset, and the specific modules formed by the two DNBs in the critical points signal the emergence of the critical transitions of the sudden changes for the disease. For the validation of disease onset time points, the many evidence and public reports were used to validate the onset of T1D in the NOD mouse, and in particular, we found that the reports of disease onset for T1D in the NOD mouse were consistent with the disease onset time points the DNBs marked. Hence, DNBs can be used to detect the early-warning signals of T1D, predict upcoming disease onset before the phenotype occurrence. DNB can also be adopted to analyze the molecular mechanism of the disease initiation and progression of the disease based on the identified leading networks.

## Competing interests

The authors declare that they have no competing interests.

## Authors' contributions

LC designed research. XL, RL and XMZ performed data analysis. XL and CL wrote the paper. All authors read and approved the final manuscript.

## Supplementary Material

Additional file 1 **function annotations for the genes of the two DNBs**. The first DNB contains 95 genes, but only 85 genes can be annotated by DAVID online tool. The second DNB contains 96 genes, but only 80 genes can be annotated by DAVID online tool. Click here for file

Additional file 2 **the genes are regulated by the first DNB in next time point of the DNB appearance**. "Gene_Name" represents the gene symbol. "Gene_ID" means Entrez Gene ID. "T1D_Publication" indicates the number of T1D-specific publications associated with the gene. "In_Beta_Cell_or_Islets" indicates that the gene is expressed in beta cells/islets. "In_Mouse_Genetic_Region" indicates that the gene was found in a mouse linkage region. Click here for file

Additional file 3 **the genes are regulated by the second DNB in next time point of the DNB appearance**. "Gene_Name" represents the gene symbol. "Gene_ID" means Entrez Gene ID. "T1D_Publication" indicates the number of T1D-specific publications associated with the gene. "In_Beta_Cell_or_Islets" indicates that the gene is expressed in beta cells/islets. "In_Mouse_Genetic_Region" indicates that the gene was found in a mouse linkage region. Click here for file

Additional file 4 **the genes linked the two DNBs**. "Gene_Name" represents the gene symbol. "Gene_ID" means Entrez Gene ID. "T1D_Publication" indicates the number of T1D-specific publications associated with the gene. "In_Beta_Cell_or_Islets" indicates that the gene is expressed in beta cells/islets. "In_Mouse_Genetic_Region" indicates that the gene was found in a mouse linkage region. Click here for file

Additional file 5 **This file showed the correlation between DNB and every pathway of mouse from KEGG database**. Connection Count is the number of high correlation gene pairs between DNB and pathway. Source number is the number of DNB genes which highly correlated with some genes in pathway. Target number is the number of pathway genes which highly correlated with some genes in DNB. DNB genes count is the number of genes in this DNB. Pathway genes count is the number of genes in this pathway. Click here for file

## Declarations

The publication costs for this article were funded by the corresponding author.

This article has been published as part of *BMC Medical Genomics *Volume 6 Supplement 2, 2013: Selected articles from the Second Annual Translational Bioinformatics Conference (TBC 2012). The full contents of the supplement are available online at http://www.biomedcentral.com/bmcmedgenomics/supplements/6/S2.
